# Data Resource Profile: Climate and Enteric Diseases Research Project (ClimED)

**DOI:** 10.1093/ije/dyaf215

**Published:** 2026-01-02

**Authors:** Paul L C Chua, Lina Madaniyazi, Aurelio Tobias, Chris Fook Sheng Ng, Vera Ling Hui Phung, Rui Pan, Nasif Hossain, Rosana Abrutzky, Gabriel Carrasco Escobar, Dung T Phung, Abu Syed Golam Faruque, Patrick Brown, Micheline de Sousa Zanotti Stagliorio Coêlho, Paulo Hilario Nascimento Saldiva, Eric Lavigne, Miguel Antonio Salazar, Dominic Royé, Chau-Ren Jung, Kraichat Tantrakarnapa, Wissanupong Kliengchuay, Noah Scovronick, Victoria Lynch, Jinah Park, Yoonhee Kim, Cunrui Huang, Jan C Semenza, Simon Hales, Masahiro Hashizume

**Affiliations:** Department of Global Health Policy, Graduate School of Medicine, University of Tokyo, Bunkyo-ku, Tokyo 113-0033, Japan; Department of Global Health, School of Tropical Medicine and Global Health, Nagasaki University, Sakamoto, Nagasaki 852-8523, Japan; Department of Global Health, School of Tropical Medicine and Global Health, Nagasaki University, Sakamoto, Nagasaki 852-8523, Japan; Institute of Environmental Assessment and Water Research, Spanish Council for Scientific Research, Barcelona 08034, Spain; Department of Global Health Policy, Graduate School of Medicine, University of Tokyo, Bunkyo-ku, Tokyo 113-0033, Japan; Department of Global Health Policy, Graduate School of Medicine, University of Tokyo, Bunkyo-ku, Tokyo 113-0033, Japan; Department of Global Health Policy, Graduate School of Medicine, University of Tokyo, Bunkyo-ku, Tokyo 113-0033, Japan; University of Virginia Environmental Institute, Charlottesville, Virginia 22908, United States; Gino Germani Research Institute, Faculty of Social Sciences, University of Buenos Aires, Buenos Aires C1053ABH, Argentina; Health Innovation Laboratory, Institute of Tropical Medicine Alexander von Humboldt, Cayetano Heredia University, Lima 15102, Peru; School of Public Health, The University of Queensland, Herston, Queensland 4006, Australia; Nutrition Research Division, International Centre for Diarrhoeal Disease Research Bangladesh, Dhaka 1212, Bangladesh; Centre for Global Health Research, St Michael’s Hospital, Toronto, Ontario M5B 1W8, Canada; Department of Statistical Sciences, University of Toronto, Toronto, Ontario M5G 1ZS, Canada; Department of Pathology, Faculty of Medicine, University of São Paulo, São Paulo 01246-903, Brazil; Department of Pathology, Faculty of Medicine, University of São Paulo, São Paulo 01246-903, Brazil; School of Epidemiology and Public Health, Faculty of Medicine, University of Ottawa, Ottawa, Ontario K1G 5Z3, Canada; Environmental Health Science and Research Bureau, Health Canada, Ottawa, Ontario K1A 0K9, Canada; Graduate School, Angeles University Foundation, Angeles City, Pampanga 2009, Philippines; The Biological Mission of Galicia, Spanish Council for Scientific Research, Salcedo, Pontevedra 36143, Spain; Department of Public Health, College of Public Health, China Medical University, Taichung 40402, Taiwan R.O.C; Department of Social and Environmental Medicine, Faculty of Tropical Medicine, Mahidol University, Bangkok 10400, Thailand; Environment, Health and Social Impact Unit, Faculty of Tropical Medicine, Mahidol University, Bangkok 10400, Thailand; Department of Social and Environmental Medicine, Faculty of Tropical Medicine, Mahidol University, Bangkok 10400, Thailand; Environment, Health and Social Impact Unit, Faculty of Tropical Medicine, Mahidol University, Bangkok 10400, Thailand; Department of Environmental Health, Rollins School of Public Health, Emory University, Atlanta, Georgia 30322, United States; Department of Environmental Health Sciences, Mailman School of Public Health, Columbia University, New York 10027, United States; Department of Public Health Sciences, Graduate School of Public Health, Seoul National University, Seoul 08826, South Korea; Department of Global Environmental Health, Graduate School of Medicine, The University of Tokyo, Bunkyo-ku, Tokyo 113-0033, Japan; Vanke School of Public Health, Tsinghua University, Beijing 100084, China; Department of Epidemiology and Global Health, Umeå University, Umeå, SE-90187, Sweden; Heidelberg Institute of Global Health, University of Heidelberg, Heidelberg 69120, Germany; Department of Public Health, University of Otago, Wellington 6242, New Zealand; Department of Global Health Policy, Graduate School of Medicine, University of Tokyo, Bunkyo-ku, Tokyo 113-0033, Japan; Department of Global Health, School of Tropical Medicine and Global Health, Nagasaki University, Sakamoto, Nagasaki 852-8523, Japan

**Keywords:** diarrhoea, gastroenteritis, climate, weather, dataset

Key Features

- The Climate and Enteric Diseases Research Project features a dataset that contains time series of enteric-disease cases and climate-related exposures, organized by week and at a subnational administrative level.
- Processed enteric-diseases data include deaths, hospital admissions and visits, and surveillance cases with pathogen-specific breakdown from 49 nations with 1290 subnational locations spanning >20 years starting from the year 2000.
- Climate-related exposures include 2-metre temperatures, total precipitation, as well as tropical cyclone maximum sustained winds and total rainfall, which were uniformly extracted and processed by using bespoke R functions, collected from the European Centre for Medium-Range Weather Forecasts Reanalysis 5th Generation Land and Inter-Sectoral Impact Model Intercomparison Project 3a datasets.
- Requests for the processed data can be made via https://paulcarlos.quarto.pub/climed/, subject to approval from each data provider.

## Data resource basics

Enteric diseases, also known as diarrhoeal or intestinal infectious diseases in humans, are a significant public health problem worldwide, especially in low- and middle-income countries, in which the majority of the burden still exists [1]. Climate change can impact the global burden of enteric diseases because changes in weather and other major climate-related events can alter the transmission of enteropathogens, which in turn can indirectly increase morbidity and mortality. Existing literature suggests that warmer temperatures, heavy rainfall, and flooding may increase the risk of enteric-disease morbidity [2–4]. Given the projected rise in temperatures, variability in rainfall (e.g. more days with extreme rainfall or very little rainfall), increased flooding occurrences, and intensification of tropical cyclones, severe enteric-disease morbidity and mortality related to climate change are likely to occur in the future [5].

Although numerous studies have analysed the impacts of climate and climate-related events on enteric diseases, a significant limitation is deriving global- or regional-level estimates for enteric risk due to climate exposures, mainly due to the substantial heterogeneity when pooling risk curves across studies in the literature [2, 3]. This is because each study has varying definitions of exposure and outcome, modelling approaches, and spatiotemporal resolutions. To overcome this limitation, the Climate Change and Enteric Diseases Research (ClimED) was established to consolidate multi-country human diarrheal disease datasets of varying types (e.g. mortality and morbidity) together with processed climate-related variables from open data sources. The consolidated dataset incorporates standardized definitions of exposure and outcome, enabling the application of a uniform modelling approach to generate consistent risk curves across different regions, as well as pooled risk estimates. These pooled risk curves can then be used to estimate both historical and projected impacts of climate change on diarrheal diseases, while accounting for adaptation and mitigation measures. ClimED was initiated by the Department of Global Health Policy at the University of Tokyo in May 2023 and they coordinated with various international collaborators who have access to datasets on enteric diseases from governmental health records, surveillance systems, and national health-insurance databases.

## Data collected

**Figure 1.**
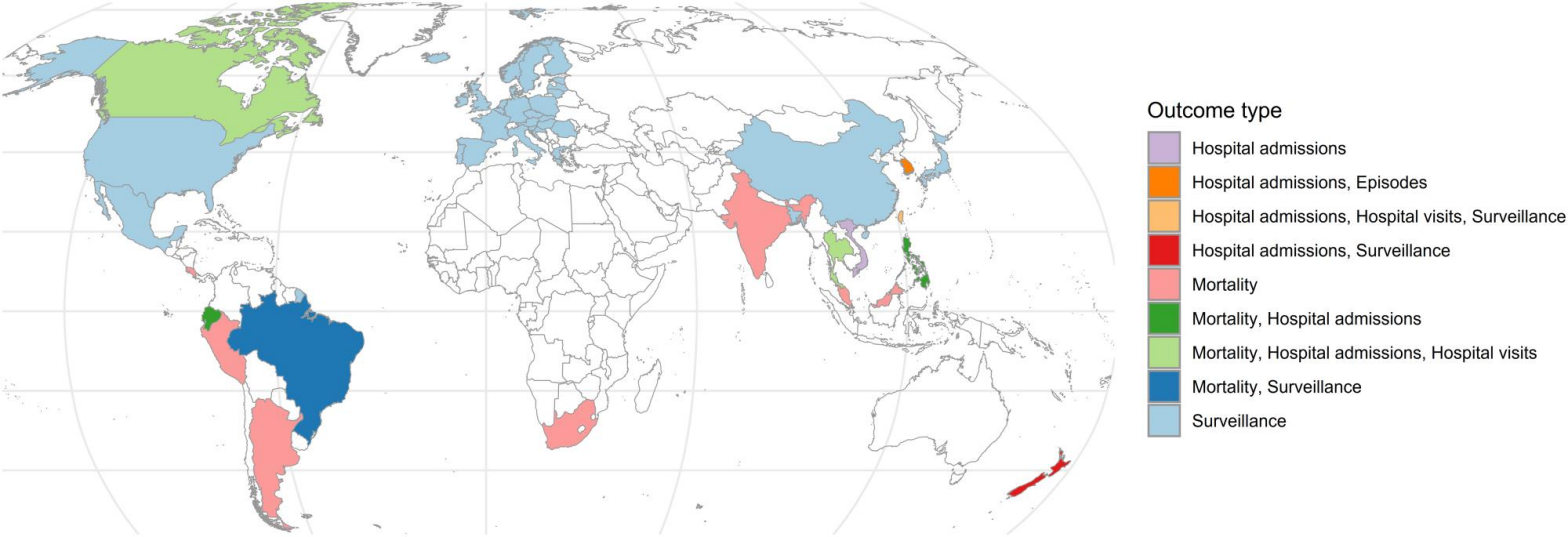
Countries and territories with enteric-disease datasets.

**Figure 2.**
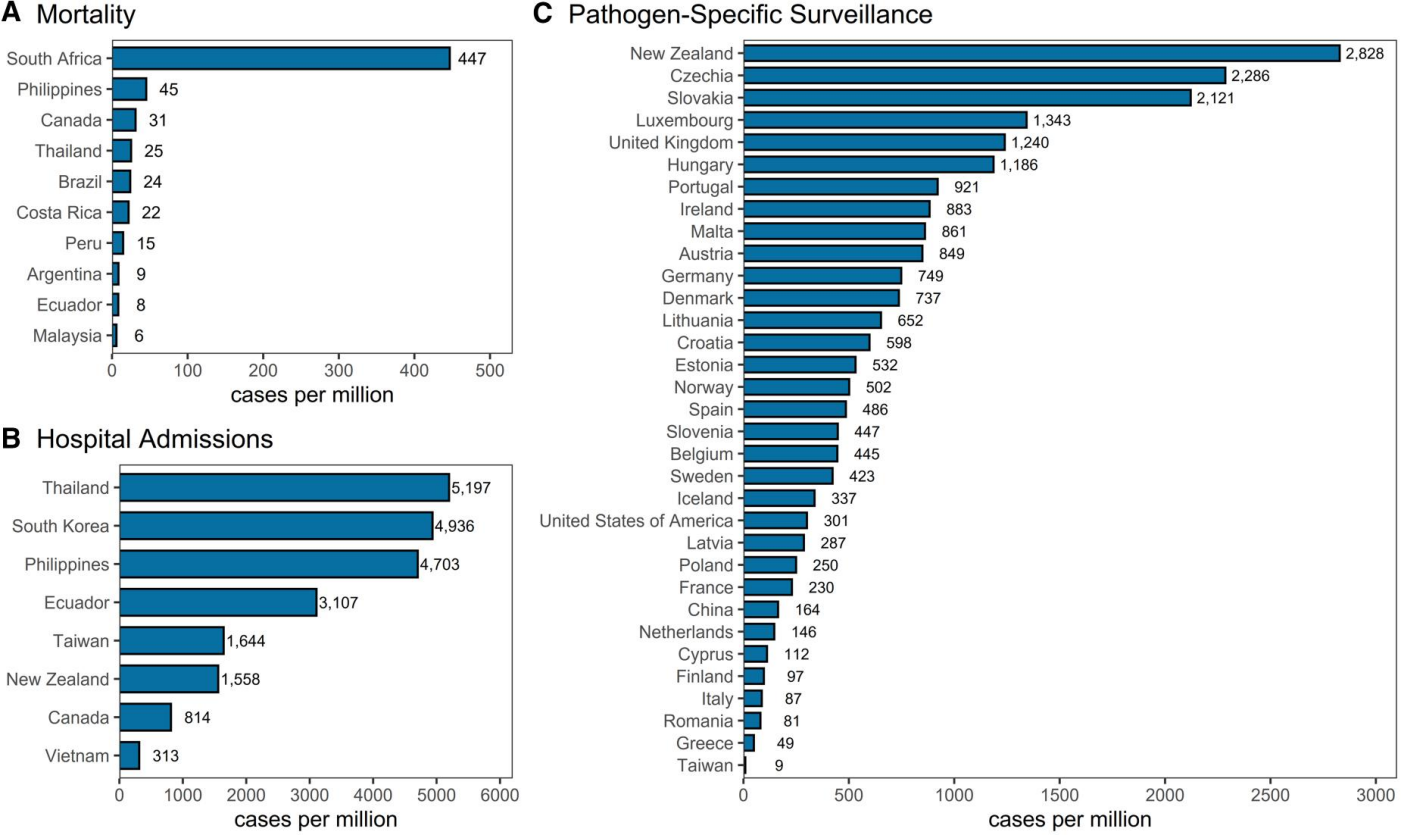
Rates of enteric diseases per million individuals per year based on gathered datasets.

**Figure 3.**
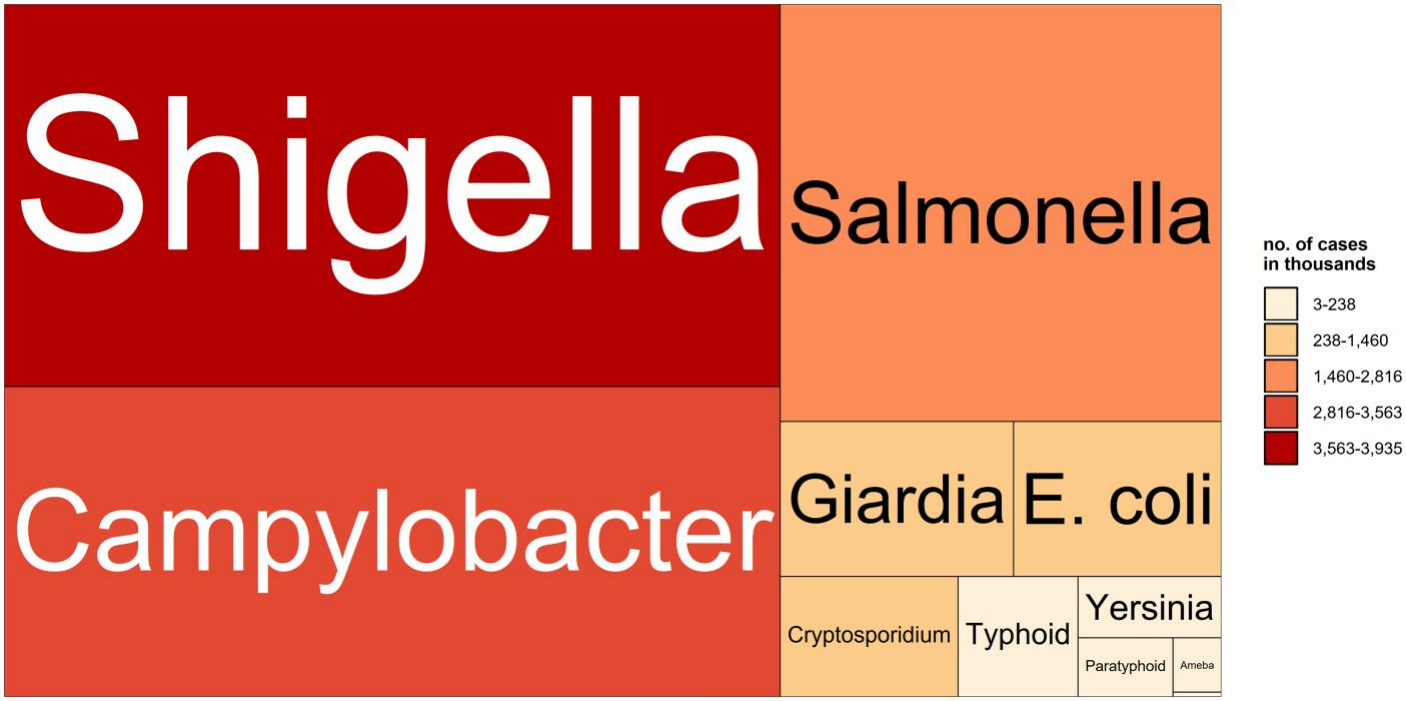
Pathogens included in the collected datasets.

For the data on enteric diseases, we compiled a total of 664 501 mortality counts and 407 613 314 morbidity counts. Morbidity cases further comprise 106 509 550 episodes (i.e. sum of all types of outcomes reported by South Korean National Health Insurance Service), 12 958 433 hospital admissions, 112 575 267 hospital visits (i.e. emergency room and outpatient visits), and 185 773 057 surveillance cases in 49 countries and territories with 1290 subnational units from 1993 to 2024 (Figs 1 and 2). We included enteric-diseases data that are coded or categorizable under the 10th revision of the International Classification of Diseases (ICD10) A00–A09, which are intestinal infectious diseases. The mortality data are collected from the national statistical offices of Argentina, Brazil, Canada, Costa Rica, Ecuador, Malaysia, Peru, Philippines, South Africa, and Thailand. For India, mortality data are collected from the Million Death Study—a nationally representative survey undertaken by the Registrar General of India’s Sample Registration System [6]. The hospital data from Bangladesh and Vietnam are directly collected from major hospitals, whereas the data from the Philippines and South Korea are collected from their national health-insurance databases. The remaining morbidity data are collected from national surveillance systems or governmental databases of the respective countries and territories. Pathogen-specific cases were available from surveillance systems of upper-middle- and high-income countries (Fig. 3). *Shigella* cases (i.e. bacillary dysentery) are the highest in number, with the majority from Mainland China [7]. Other major pathogens, such as *Campylobacter* and *Salmonella*, were identified through surveillance systems in high-income countries, including European countries, the USA, and New Zealand. Some pathogens were identifiable from ICD-10 codes for hospital admissions and visits. Some datasets have age and sex information. Full details of the raw datasets from each country are listed in https://paulcarlos.quarto.pub/climed/.

**Figure 4.**
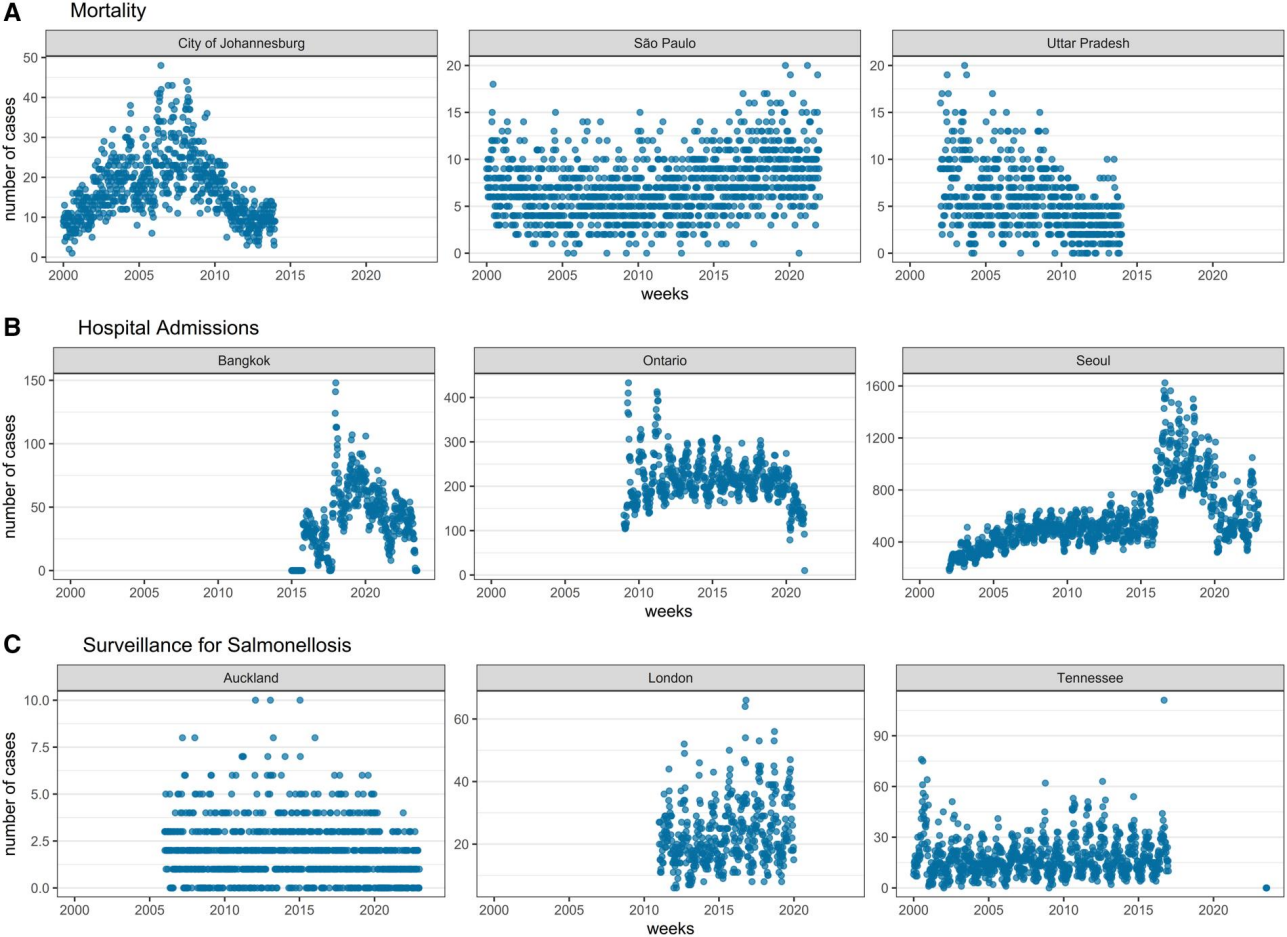
Weekly time series of enteric-disease cases by data source type and selected subnational units.

**Figure 5.**
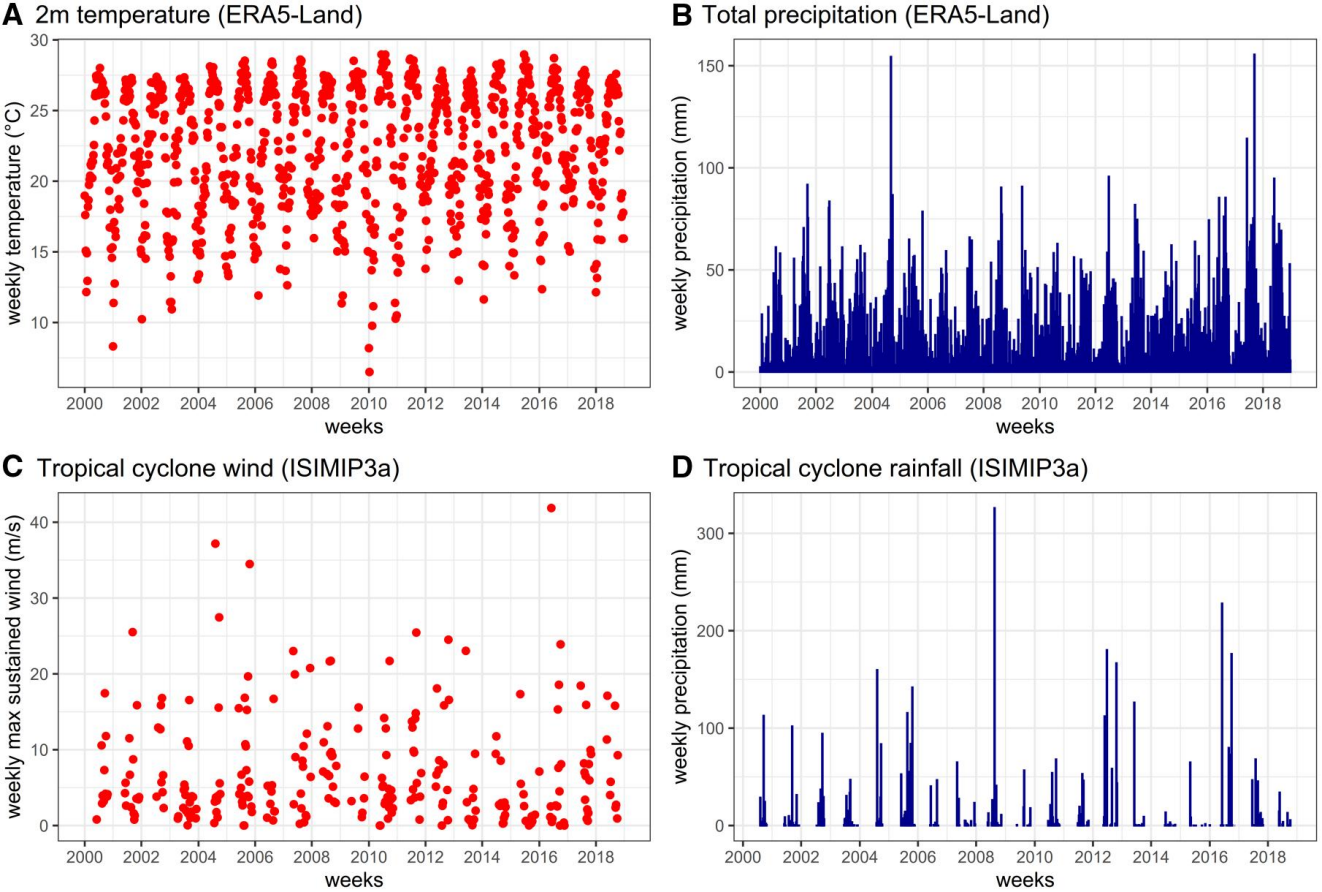
Weekly time series of climate-related variables in the State of Florida, USA.

The raw datasets were directly processed into weekly time series by subnational units (Fig. 4). The weekly temporal resolution was deemed appropriate as the majority of datasets were reported at weekly intervals. Cases recorded from 2000 onward were retained, while earlier cases were excluded to focus on the most recent two decades since the start of the new millennium. Most datasets were processed in the International Organization for Standardization (ISO) week format, while a few followed the epidemiological (EPI) week format (i.e. Mexico, the USA, and Taiwan). These weekly formats were selected to ensure a consistent 7-day aggregation of cases. The subnational units per country were chosen based on the smallest possible administrative units that yielded fewer zero weekly cases. The shapefiles or polygons of administrative boundaries for each subnational unit were collected from official government websites (e.g. statistical offices) or open sources, such as the Humanitarian Data Exchange by the United Nations Office for the Coordination of Humanitarian Affairs and Integrated Public Use Microdata Series. Temperatures and total precipitation from the European Centre for Medium-Range Weather Forecasts Reanalysis 5th Generation Land (ERA5-Land) (i.e. land component of the fifth generation of European ReAnalysis) [8] and maximum sustained winds and rainfall from tropical cyclones from Inter-Sectoral Impact Model Intercomparison Project 3a (ISIMIP3a) (i.e. the first component of the ISIMIP) [9] based on wind field models [10, 11] and a rainfall algorithm [12] were extracted and processed by following the temporal and spatial format of the enteric-diseases data. Population density from Gridded Population of the World version 4.11 and climate zones from Beck *et al.* (2023) [13] were also extracted and processed for each subnational unit (Fig. 5). Bespoke R functions, which are a wrapper of R functions from the base, terra [14], ncdf4 [15], lubridate [16], and lutz [17] R packages, were created to uniformly extract the population-weighted averages of all climate-related variables by subnational units following their respective time zones (see https://github.com/paulcarlos/climed_codes). Our R functions can also process data from ERA5 as an alternative source of climate variables. Table 1 enumerates the format of the processed data.

**Table 1.** Format of the processed-weekly time-series data.

Variable name	Description
name	Name of country
subnat	Name of subnational administrative unit
yrwk	Contains year and number of week following either the ISO or EPI week format
outcome	Type of dataset, e.g. mortality, hospitalizations, hospital visits, and surveillance
pathogen	Either ‘all-cause’ or name of pathogen if available
cases	Total weekly counts of enteric-disease cases
t2m	Population-weighted average of 2-metre temperatures in degrees Celsius at ∼9-km grid resolution of ERA5-Land [8]
tp	Population-weighted average of total precipitation (water and snow) in millimetres at ∼9-km grid resolution of ERA5-Land
tcwind	Population-weighted maximum sustained wind speed in metres per second from tropical cyclones at 10-km grid resolution of ISIMIP3a [9] based on the wind field model of Emanuel and Rotunno (2011) [10]
tcrain	Population-weighted total rainfall in millimetres from tropical cyclones at 10-km grid resolution of ISIMIP3a based on Zhu *et al.* (2013) [12]
popden	Population density in individuals per kilometre derived from Gridded Population of the World version 4.11
climzone	Climate zone based on Beck *et al.* (2023) Koppen–Geiger classification map [13]

## Data resource use

The processed-weekly ClimED dataset is primarily designed for modelling the relationship between climate-related exposures and enteric diseases by using widely adopted methods such as time-series analysis or time-stratified case-crossover, both of which have been shown to produce robust exposure–outcome risk curves [18, 19]. The dataset can be used to examine various relationships between climate-related exposures and specific enteric-disease outcomes. For example, the temperature sensitivity of enteric diseases may depend on the type of registered outcome, such as hospitalization versus mortality [20]. Moreover, differences in the climate sensitivity of enteric diseases can be analysed in terms of their spatial features. For example, ClimED datasets have been used to create risk curves for total precipitation and enteric-disease mortality by major climate zones [21]. The generated risk curves can be applied to calculate the historical and future numbers of all-cause or pathogen-specific enteric-disease cases attributable to climate change.

Several enteric-disease datasets include additional information on age, sex, and relatively small administrative units in which each case/patient was reported (e.g. 3600, 3400, and 1600 Chinese, Brazilian, and Philippine municipalities, respectively). Using these details, age- and sex-specific climate sensitivities can be tested. Additionally, spatial analysis and downscaling can be performed by using datasets with finer spatial resolutions.

## Strengths and weaknesses

The strength of the ClimED dataset is its spatiotemporal dimensions. The multi-location and multi-country coverage enables the generation of outputs applicable to various categories of locations (e.g. by climate or by income level) or according to geographical regions, such as Asia, America, and Europe. The rich temporal dimension allows the examination of both short- and long-term associations through year-long or seasonal analyses, which is essential for climate-related research. Another strength of the ClimED dataset is its extraction of several climate variables from gridded open-source datasets that are not easily accessible to public health researchers. In addition to the few climate variables currently included, the extraction codes can be applied to various gridded datasets to generate exposure variables consistent with the spatiotemporal dimensions of the enteric-disease data.

Weaknesses of the dataset include:

- Spatial resolutions vary across countries, with some available at the national level and others at large subnational units (e.g. states in the USA). Additionally, not all country datasets start in the year 2000 and they have varying lengths or numbers of years (see Fig. 4). These limit the comparability of outputs from each country.
- Morbidity data vary across countries because some are collected directly from hospitals (i.e. admissions or visits) or health-insurance databases, while the rest are from surveillance systems. The surveillance systems also vary across countries, with most collecting data on specific pathogens (e.g. food-borne) and some collecting data from sentinel sites or healthcare facilities. The data user should consider these aspects to ensure proper alignment with their conceptual framework or mechanisms being tested.
- Weekly time series were directly aggregated from raw datasets and were not validated or corrected for their apparent flaws, such as outliers and sudden fluctuations (see Fig. 4). A data user should review each time series carefully and make necessary corrections or adjustments to suit their analysis, ensuring a proper fit in statistical models.
- Pathogen-specific datasets were either obtained from surveillance systems or identified through ICD-10 codes in hospital or health-insurance claims records. Surveillance systems are consistently testing for pathogens, unlike healthcare institutions, which may not regularly test for pathogens that cause enteric infections. A data user should be aware of these differences when building their models or analysing pathogen-specific data.
- The extracted climate variables are from modelled datasets and the values represent the average of all grids or cells that fall within a subnational unit. This is not the same as weather-station data, which report observed or actual values within a small space. For example, the rainfall data by grid are smaller in value compared with weather-station data because the water or rainfall was modelled to spread evenly across a 9-km grid square. A study reported that ERA5-Land produces temperature–mortality risk curves comparable to those from weather stations, albeit with some underestimation in tropical regions [22]. Total precipitation from ERA5-Land is subject to biases and limitations compared with observed values from rain gauges, which vary depending on the area and conditions [23–25]. Tropical cyclone rainfall was derived from ERA5.

## Data resource access

The ClimED dataset is not publicly accessible because the enteric-diseases data follow privacy rules from primary data sources. Individuals can request the data through the website (https://paulcarlos.quarto.pub/climed/) or e-mail correspondence to paulchua@m.u-tokyo.ac.jp to facilitate data collection across primary data providers. The approval for data release will be based on the decision of each primary data provider.

## Ethics approval

Ethics approval is not applicable because all enteric-disease data exclude personal identifiers and are aggregated by week and into intermediate- to large-scale subnational units, and the gridded climate and population data were collected from open sources. Only ClimED members have direct access to the processed datasets.

## Data Availability

All R and Python code for data processing and for creating the ClimED website is available at https://github.com/paulcarlos/. Processed time-series datasets of enteric-disease cases, matched with climate-related exposure data, can be requested at https://paulcarlos.quarto.pub/climed/, subject to the approval of the data providers.
